# 201. Comparison of Bloodstream Infections Between Hospitalized Patients with and without COVID-19 Infection During the First Wave of the COVID-19 Pandemic in a Community Hospital in South Bronx: An Observational Study

**DOI:** 10.1093/ofid/ofab466.403

**Published:** 2021-12-04

**Authors:** Afsheen Afzal, Edgar Gomez, Victor Perez Guttierrez, Aye Myat Mon, Carolina Moreira Sarmiento, Amna Khalid, svetlana Polishchuk, Mohannad Al-Khateeb Al-Khateeb, Mubarak Yusuf, Boyana Yankulova, Yinelka Silverio De Castro, Anjana Pillai, Usha Venugopal, Addi Feinstein, Alexander LaFortune, Daniel Sittler, Karen Hennessey, Vidya Menon

**Affiliations:** Lincoln Medical Center, New York, New York

## Abstract

**Background:**

Comparative data on bloodstream infections (BSI) in hospitalized patients with and without SARS-CoV2 positive test is lacking.

**Methods:**

A retrospective observational study comparing (BSI) with and without COVID-19 infection was performed was performed from Jan1- May 1, 2020. Patient demographics, clinical microbiological characteristics of infections, therapeutic interventions and outcomes was compared between the two groups.

**Results:**

Of 155 patients with BSI, 104 were SARS-CoV2 PCR negative (N) while 51 were positive (Table 1). Majority of SARS-CoV2 positives (P) had ARDS (58.8%), required mechanical ventilation (73%), inotropic support (55%), therapeutic anticoagulation (28%), proning (35%), Rectal tube (43%), Tocilizumab (18%), and steroids (43%) (Table 2). BSI was higher in N with HIV (16.3% vs 3.9% p=0.027). Duration of antibiotic therapy (DOT) prior to BSI was significantly longer in P (15 days vs. 5 days, p < 0.0001) (table 2). In-hospital mortality was significantly higher among P with BSI (49% vs. 21% p < 0.0001). 185 BSI events were observed during the study period with 117 in N patients and 68 in P. Primary BSI was predominant (76%) in N while secondary BSI (65%) was common in P of which 50% were CLABSI. Median time from admission to positive culture was 0.86 days in N compared to 12.4 in P (p = 0.001). Majority of BSI in P were monomicrobial (88%) and hospital acquired (71%) when compared to N (p< 0.001). *Enterococcus spp* (28%), *Candida spp*(12%), MRSA (10%) and *E.coli* (10%) were predominant microbes in P compared to Streptococcus grp (16%), MSSA (14%), MRSA (13%) and *E.coli* (12%) in N (figure 1). Mortality from BSI was associated with COVID-19 infection (OR 2.403, p = 0.038), DM (OR 2.335, p = 0.032), Charlson comorbidity index >3 (OR 1.236, p = 0.004), and mechanical ventilation (OR 11.398, p < 0.001) on multivariate analysis.

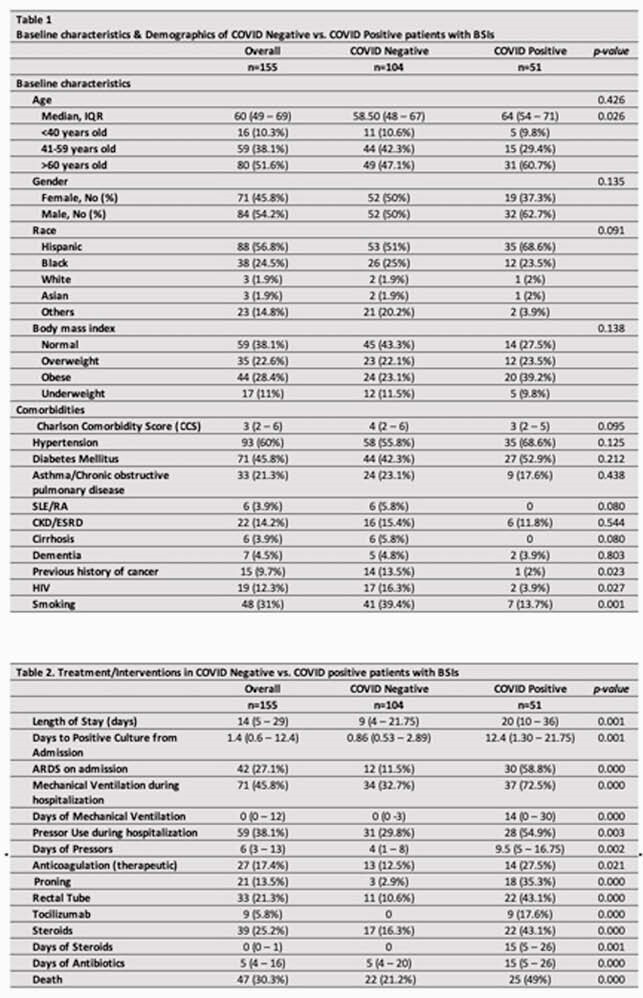

Comparison of Microorganisms isolated in the BSI

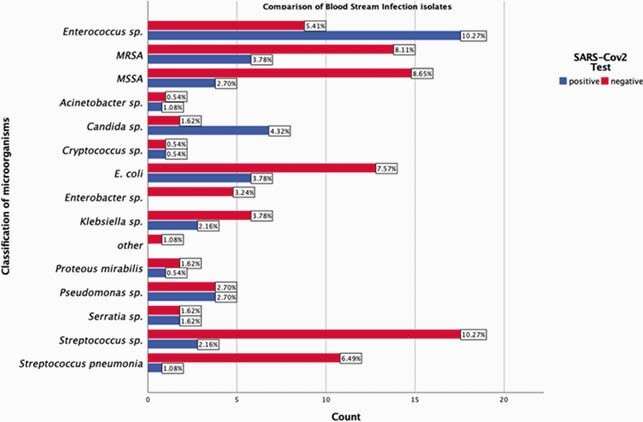

X-axis represents the number of BSI events whereas the number at the end of each bar represents the percentage

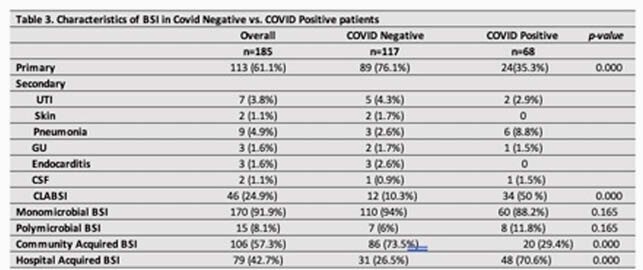

**Conclusion:**

Increased events of hospital acquired, secondary BSI (CLABSI) due to E*nterococcus* was observed in adult P compared to N. These patients were critically ill, developed BSI in the second week of hospitalization, had longer DOT prior to positive cultures and worse outcomes. Breakdown of infection control measures and inappropriate antimicrobial use during the surge could be contributory.

**Disclosures:**

**All Authors**: No reported disclosures

